# Postsurgical Stability of Temporomandibular Joint of Skeletal Class III Patients Treated with 2-Jaw Orthognathic Surgery via Computer-Aided Three-Dimensional Simulation and Navigation in Orthognathic Surgery (CASNOS)

**DOI:** 10.1155/2021/1563551

**Published:** 2021-08-06

**Authors:** Ling-Chun Wang, Yi-Hao Lee, Chi-Yu Tsai, Te-Ju Wu, Ya-Ying Teng, Jui-Pin Lai, Shiu-Shiung Lin, Yu-Jen Chang

**Affiliations:** ^1^Department of Craniofacial Orthodontics, Department of Dentistry, Kaohsiung Chang Gung Memorial Hospital and Chang Gung University College of Medicine, Kaohsiung, Taiwan; ^2^Department of Craniofacial Orthodontics, Chang Gung Memorial Hospital, Linkou, Taiwan; ^3^Graduate Institute of Craniofacial and Dental Science, Chang Gung University, Taiwan; ^4^Department of Plastic and Reconstructive Surgery, Kaohsiung Chang Gung Memorial Hospital and Chang Gung University College of Medicine, Kaohsiung, Taiwan

## Abstract

**Objective:**

The aim of this study is to clarify the postsurgical stability of temporomandibular joints in skeletal class III patients treated with 2-jaw orthognathic surgery which was performed utilizing computer-aided three-dimensional simulation and navigation in orthognathic surgery (CASNOS) protocol.

**Materials and Methods:**

23 consecutive nongrowing skeletal class III patients with mandibular prognathism associated with maxillary retrognathism treated with 2-jaw orthognathic surgery between 2018 and 2019 were enrolled in this study. The surgery was planned according to the standardized protocol of CASNOS (computer-aided three-dimensional simulation and navigation in orthognathic surgery). Computed tomography (CT) scans were performed in all patients 3 weeks presurgically and 6 months postsurgically. ITKSNAP and 3D Slicer software were used to reconstruct three-dimensional facial skeletal images, to carry out image segmentation, and to superimpose and quantify the TMJ position changes before and after surgery. Amount of displacement of the most medial and lateral points of the condyles and the change of intercondylar angles were measured to evaluate the postsurgical stability of TMJ.

**Results:**

A total amount of 23 skeletal class III patients (female : male = 12 : 11) with age ranged from 20.3 to 33.5 years (mean: 24.39 ± 4.8 years old) underwent Le Fort I maxillary advancement and BSSO setback of the mandible. The surgical outcome revealed the satisfactory correction of their skeletal deformities. The mean displacement of the right most lateral condylar point (RL-RL′) was 1.04 ± 0.42 mm and the mean displacement of the left most lateral condylar point (LL-LL′) was 1.19 ± 0.41 mm. The mean displacement of the right most medial condylar point (RM-RM′) was 1.03 ± 0.39 mm and the left most medial condylar point (LM-LM′) was 0.96 ± 0.39 mm. The mean intercondylar angle was 161.61 ± 5.08° presurgically and 159.28 ± 4.92° postsurgically.

**Conclusion:**

The postsurgical position of TM joint condyles in our study only presented a mild change with all the landmark displacement within a range of 1.2 mm. This indicates the bimaxillary orthognathic surgery via 3D CASNOS protocol can achieve a desired and stable result of TMJ position in treating skeletal class III adult patients with retrognathic maxilla and prognathic mandible.

## 1. Introduction

Structural changes of the condyles may occur after orthognathic surgeries due to the adaptation mechanism after mandibular osteotomies which lead to the changes of loading distribution [1]. It can be classified into two categories of condylar structural changes as condylar remodeling and condylar resorption [2, 3]. The former is a physiological process, and the latter is a pathological change. Clinical symptoms of temporomandibular joint (TMJ) and relapse of surgical outcome may follow after condylar resorption.

Another issue associated with the postsurgical stability of the TMJ condyles is the alteration of their position after orthognathic surgery which often occurs after mandibular osteotomies [1]. Some studies believed that several complications after orthognathic surgery such as condylar resorption, disc displacement, and other symptoms of temporomandibular joint disorders (TMD) may be associated with the significant position change of the condyles [4, 5]. The relationship between orthognathic surgery and TMD is still poorly understood, and the acceptable and harmless amount of condylar position change remains unclear. Previous studies regarding the alteration of condylar position were frequently analyzed with 2D radiographs or the slicing images in 3D radiographs [6–10]. Furthermore, the condyle-fossa relationship was often assessed with 2D measurement. The analysis utilizing three-dimensional imaging system and the actual amount of the condylar position changes were rarely shown.

In our study, we applied 3D imaging software to reconstruct the craniofacial area from the preoperative and postoperative data of computer tomography. The superimposition of two-stage 3D image and quantitative measurement was carried out. It was aimed at investigation of postsurgical stability of TMJ position in skeletal class III patients treated with 2-jaw surgery using the standard protocol of CASNOS (computer-aided three-dimensional simulation and navigation in orthognathic surgery).

## 2. Materials and Methods

The retrospective study was carried out on computed tomography (CT) scans of nongrowing class III skeletal patients with mandibular prognathism and maxillary retrognathism, who received nonextraction orthodontic and orthognathic treatment including Le Fort I osteotomy combined bilateral sagittal split osteotomy (BSSO). The enrolled patients were treated between July 2018 and December 2019 at the Craniofacial Center, Kaohsiung Chang Gung Memorial Hospital. The selected criteria for the skeletal class III patients were corresponded: overjet ≤ −5 mm; ANB ≤ 0 degree [11]. The exclusion criteria were those patients who presented with degenerative TMJ disease, severe facial asymmetry, deformity secondary to trauma, cleft lip and palate, or systemic disease. Treatment with extraction pattern was also excluded. All operations were arranged only when no further growth of patients was demonstrated, and it was assessed by superimposition of lateral cephalograms between initial and at least 6 months after presurgical orthodontic treatment.

All operations were conducted by an experienced surgeon after completion of presurgical orthodontic preparation. Before the operation, three-dimensional surgical simulation and navigation were executed according to CASNOS protocol proposed by Chang [12]. Under 3D simulation of volumetric data combining with physical manipulation of stereolithographic models and the following lab work, including the fixation miniplate, mandibular positioning splint and the occlusal splint were fabricated. During the operation, the relative positioning of the maxilla and mandible was achieved and maintained with the occlusion stent. The maxillomandibular complex was repositioned according to the planned navigation and fixed to the basal bone with the prefabricated miniplates. The fixation methods for all our orthognathic surgical patients were (1) internal fixation miniature titanium bone plates and cortical screws and (2) the intermaxillary fixation (IMF) with concomitant fixed orthodontic appliances and supplementary elastics for stabilization at least 2 weeks after the surgery.

CT images (Toshiba Aquilion 64: 120 kVp, 350 mA, rotation time: 0.5 sec, 64 × 0.5 mm slices) over the craniofacial area were obtained 3 weeks before surgery (T1) when all the required orthodontic preoperative movements had been completed. The second CT scan was obtained at 6 months postoperatively (T2) to assess the treatment outcome with orthodontic fixed appliance still in place. Two open-source software programs, ITKSNAP (available at: http://www.itksnap.org/pmwiki/pmwiki.php) and 3D Slicer (available at: http://www. http://slicer.org/), were used to precisely segment, superimpose, and quantify the TMJ position changes after surgery. Open-source software tools were applied to calculate the dental and skeletal changes. Intrarater reliability was also validated.

### 2.1. 3D Analysis of TMJ Stability

**Figure 1:**
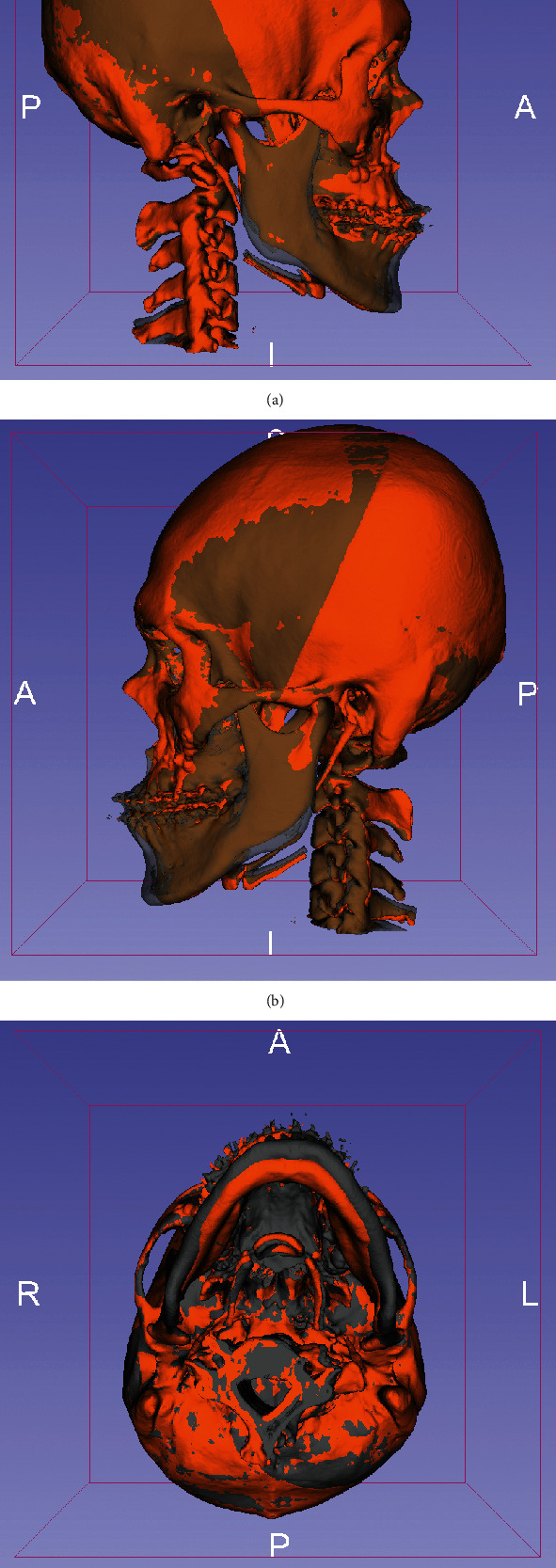
The superimposition of presurgical (T1, gray area) and postsurgical craniofacial area (T2, orange area). The head was orientated relatively to the Frankfort horizontal (FH) plane which was established by bilateral orbitale and the landmark in the middle of the two porions (mid-Po). The superimpositions of T1 and T2 scans were registered to the cranial base using a voxel-based registration algorithm ((a) the right side; (b) the left side; (c) the bottom view side).

The selected landmarks were identified using the CT images. The head orientation relative to the Frankfort horizontal (FH) plane was considered the horizontal reference. The porion (Po) and orbitale (Or) were utilized to set up the horizontal reference line. And this reference line was applied to form the horizontal reference plane with orbitale of the left side. The FH plane was formed by three points: orbitale left, orbitale right, and a landmark in the middle of the two porions (mid-Po). Landmark identification was conducted by one trained and calibrated operator, and measurements were taken by the same examiner (Ling-Chun Wang). These landmarks were identified on both the T1 (3 weeks before surgery) and the T2 (6 months after surgery) scans. All T1 and T2 scans were registered to the cranial base using a voxel-based registration algorithm (Figure 1) [13, 14].

**Figure 2:**
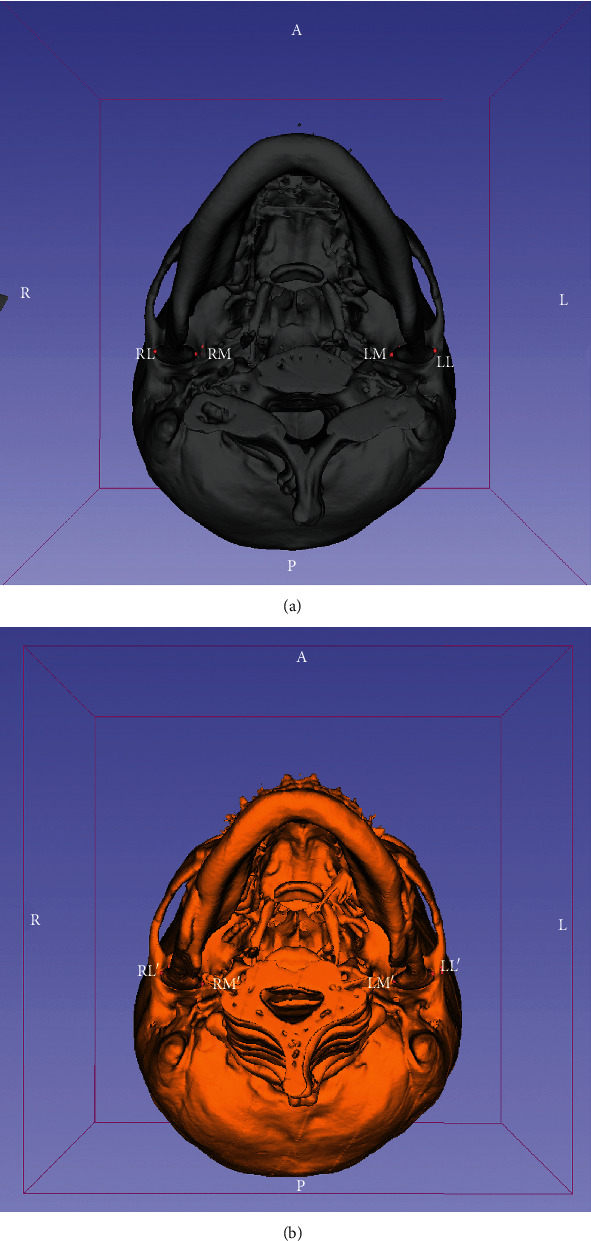
The 3D imaging of the craniofacial area reconstructed with the open-source software. RL (right), LL (left): the most lateral points of the condyles; RM (right), LM (left): the most medial points of the condyles are identified ((a) the presurgical view: T1; (b) the postsurgical view: T2).

Identification of landmarks of the TMJ (Figure 2) were defined as anatomical landmarks as (1) preoperative (T1): RL (right), LL (left)—the most lateral point of the condyle, RM (right), LM (left)—the most medial point of the condyle and (2) postoperative (T2): RL′ (right), LL′ (left)—the most lateral point of the condyle, RM′ (right), LM′ (left)—the most medial point of the condyle.

**Figure 3:**
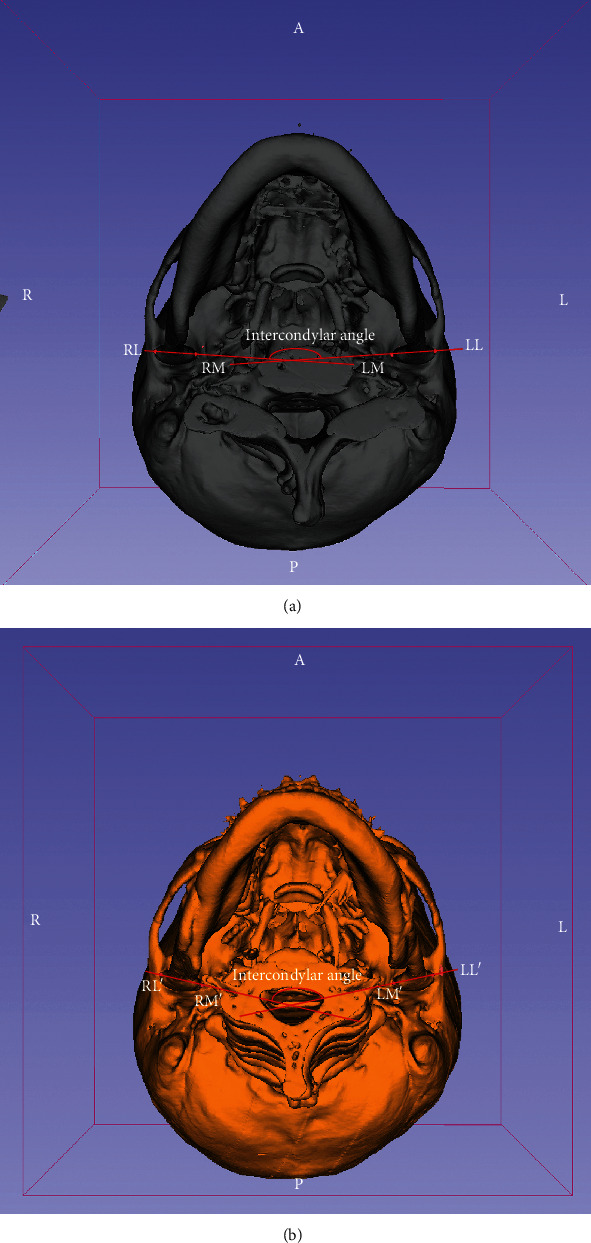
The bottom view of the mandible and cranial base. The cutting angle between the axes (intersection between RL-LM and RM-LL: intercondylar angle) was calculated and measured ((a) presurgical intercondylar angle (161.61 ± 5.08°); (b) postsurgical angle (159.28 ± 4.92°); *p* = 0.061).

All these corresponding 3D points were visualized using 3D Slicer's quantitative 3D cephalometric (quantification of 3D components [Q3DC]) tool (Figure 1). By placement of fiducial markers, this tool allows users to compute (1) the 3D distance between the T1 and registered T2 TMJ points and (2) the differences of the angle along each of the axes. Then, distances were measured between the most medial point of the condyles (RM-RM′ and LM-LM′) and between the most lateral point of the condyles (RL-RL′ and LL-LL′) in preoperative and postoperative imaging. In addition, the cutting angle between the axes (intercondylar angle) was also calculated (Figure 3). Paired *t*-test was applied to detect the differences between presurgical and 6-month postsurgical variables. The level of significance was set as the level of *p* = 0.05. The overall position discrepancy of TM joint condyles between T1 and T2 was assessed by superimposition of the frontal head surface, and the surface difference of the TMJ condyles was indicated by the color mapping that extends the discrepancies over the surface area. It was defined as the geographical summation error [12].

### 2.2. Intrarater Reliability

Intrarater reliability was measured using intraclass correlations for 3 variables (two 3D distances and intercondylar angle) in 5 subjects, with measurements taken on each subject 2 weeks apart. There was no statistical difference in defining the points and angle among the 3D quantitative points.

## 3. Results

A total of 23 patients with malocclusion who underwent bimaxillary orthognathic surgery met the eligible criteria for this study. The age of patients was ranged from 19 to 36 years (mean: 24.39 ± 4.8 years). The ratio of gender was 12 : 11 (female: male) (Table 1). The gender groups did not show any statistical difference in age. All these patients were diagnosed with midface deficiency and mandibular prognathism (Table 2), and their mean value of presurgical ANB was −6.23 ± 1.91°. The mean distances of point A and pogonion to N-perpendicular line were 0.47 ± 1.59 mm and 10.65 ± 3.73 mm, respectively. The average presurgical Wits appraisal was −11.81 ± 3.34 mm. All the patients underwent bimaxillary surgical treatment with Le Fort I maxillary advancement and BSSO setback of the mandible. The surgical outcome revealed ANB was significantly improved into 2.33 ± 1.54°; the mean distances of point A and pogonion to N-perpendicular line were 2.5 ± 1.2 mm and 1.25 ± 0.58 mm, respectively. The mean postsurgical Wits appraisal was improved into 1.32 ± 3.22 mm (Table 2). The amount of Le Fort I maxillary advancement was 3.67 ± 1.68 mm in the right side (range: 1~4.5 mm) and 3.39 ± 1.47 mm in the left side (range: 1 to 5 mm). The amount of BSSO setback of the mandible was 9.87 ± 2.51 in the right side (range: 5 to 14 mm) and 9.04 ± 2.36 mm in the left side (range: 6 to 13 mm). These average distances of maxilla advancement and distance of mandibular setback were revealed in Table 3.

The mean displacement of the right most lateral condylar point (RL-RL′) was 1.04 ± 0.42 mm, and the mean displacement of the left most lateral condylar point (LL-LL′) was 1.19 ± 0.41 mm. The mean displacement of the right most medial condylar point (RM-RM′) was 1.03 ± 0.39 mm, and the left most medial condylar point (LM-LM′) was 0.96 ± 0.39 mm (Table 4). The changes of the above targeted landmarks did not show any statistical significance between T1 and T2.

The angle between the condyles (intercondylar angle) was assessed by measuring the degrees of intersected angle formed by the two longitudinal axes of the condyles (Figures 3(a) and 3(b)). The mean angle was 161.61 ± 5.08° before and 159.28 ± 4.92° after surgery. The paired *t*-test did not reveal any significant change between the angles before and after the surgery (*p* = 0.061, Table 4).

**Figure 4:**
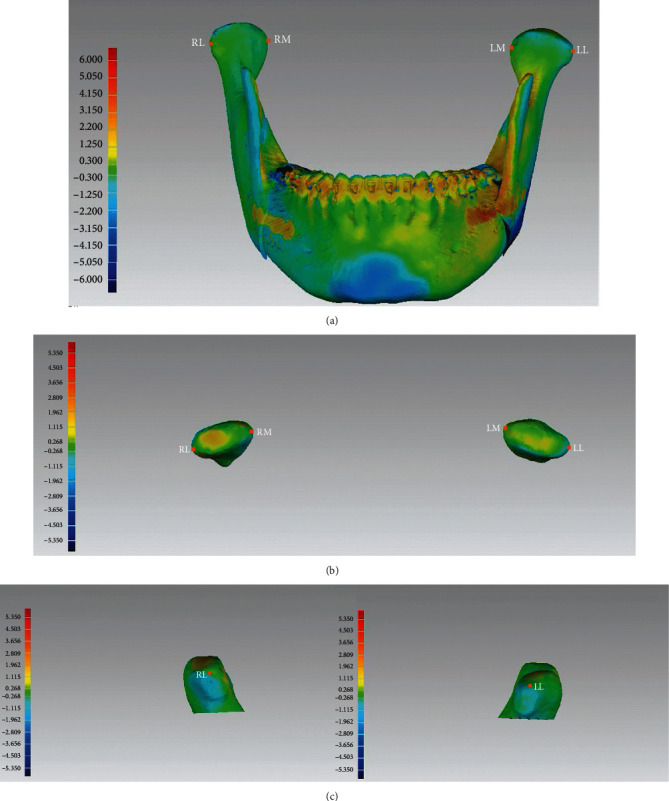
(a) The distribution of color zones indicates the means of mandibular position difference between T1 and actual T2 of the subjects. The mandibular mean differences of the patients were distributed in the green and blue zones (green: the absolute value < 0.300 mm; yellow: the absolute value < 1.250 mm). The landmarks of the most medial (RM and LM) and lateral point (RL and LL) were identified. (b) The distribution of color zones indicates the means of mandibular discrepancies on the right and left condylar heads between T1 and T2 of individual subjects. The mean discrepancies of the patients were distributed in the green and blue zones. The landmarks (RM, LM, RL, and LL) were identified from the top and lateral views. All the 3D displacements of the most lateral and medial condylar points are as follows: RL-RL′: 1.04 ± 0.42 mm; LL-LL: 1.19 ± 0.41 mm; RM-RM′: 1.03 ± 0.39 mm; and LM-LM′: 0.96 ± 0.39 mm ((b) the top view and (c) the lateral view; details in Table 4). (c) Lateral view of the condyle heads and the identified landmarks of RL and LL.

The geographical discrepancies of TMJ position between T1 and T2 were measured by calculating the summation difference of superimposition over the overall surface contour of TMJ. This geographical summation mean error was 1.43 mm ± 0.29 mm (range: 0.62 mm to 1.86 mm; Figure 4).

## 4. Discussion

The aim of this study was to assess the postsurgical stability of temporomandibular joint position in skeletal class III patients treating with 2-jaw surgery via the standard protocol of CASNOS. The accuracy of CASNOS protocol in transferring the simulation into the actual operation has been demonstrated [12]. Its benefits regarding blood loss and reduction of operation time in 2-jaw orthognathic surgery in correcting the dentoskeletal discrepancy have also been indicated [12]. All the skeletal class III patients in this study were surgically corrected into the desirable skeletal outcome which was indicated by the surgical change of ANB (from -6.23° to 2.33°) and other two linear parameters: point A and pogonion to N-perpendicular line were also improved. Point A to N-perpendicular line was corrected from 0.47 mm to 2.5 mm, and pogonion was set back from 10.65 mm to 1.25 mm relatively to the N-perpendicular line (Table 2). Nevertheless, the postsurgical stability of TM joints via 3D assessment has not yet been investigated. In this study, the position of TM joint condyles of 23 skeletal class III patients treated with combined surgical orthodontics between presurgical and 6-month postsurgical CT imaging was assessed with 3D imaging software.

In the surgical procedure of mandibular setback via bilateral sagittal split osteotomy, the proximal segments were distally moved and then fixed with the distal segments under the new designed occlusion. Under the fixation force and the vector from the temporomandibular ligaments, the condylar head rotation may occur. Several studies investigated the changes of condylar axis after mandibular osteotomies. In the previous studies, condylar axis was shown to be rotated inward in the axial view after BSSO [15–17]. However, in Katsumata's study, no obvious condylar axis rotation occurred after BSSO, but 85.9% of the condyles tended to rotate outward after IVRO [18]. Different rotation directions might be explained due to the different surgical techniques and incorporation of adjunctive procedures. In our study, no significant change between the angles of the lateral condyles before and after osteotomy was demonstrated. Our result echoes to Holzinger's study with samples treated by surgery-first orthognathic treatment [19].

The direction of immediate condylar displacement is variable. Anteroinferior, posteroinferior, and equal distributions in vertical direction were reported in the previous studies. The posterior displacement may be caused by manual manipulation over the proximal segments during the surgery, and the inferior displacement may result from intra-articular edema in the early stage after surgery [20]. Other conditions such as application of muscle relaxant under general anesthesia leading to condyle sag may also occur. After removal of surgical stent, the condyles tend to move back to the preoperative position under the force of masticatory muscles and the strain of temporomandibular ligament. With the resolution of edema, recovery change may occur [10].

The amount of condylar position change varies in each individual and is influenced by numerous factors indicated by other studies, such as surgical procedure, experience of the surgeon, and patient anatomy. In the present study, however, the position of TM joint condyles did not demonstrate any significant change postsurgically, when the recovery of masticatory function had already taken place. The stable postsurgical position of TMJ indicates the bimaxillary orthognathic surgery via 3D CASNOS protocol can achieve a desired and stable result in treating skeletal class III adult patients with retrognathic maxilla and prognathic mandible.

The findings in this study corresponded to the result in Chen et al.'s study. In Chen et al.'s study, condylar position was in a concentric position in glenoid fossa 3 months after orthognathic surgery and remained stable in one year [10]. In contrast, in the study of Harris et al., most condyles in the cases tended to displace medially, posteriorly, superiorly, and angle medially 2 months after BSSO advancement [21]. The different result might be due to the timing of assessment which was 4 months earlier than our study.

Some devices are developed for condyle stabilization during orthognathic surgery to prevent unwanted condylar movement from the original position [22]. In the present study, no such positioning device was used except the 3D surgical navigation plates which were fabricated according to the CASNOS protocol. The CASNOS protocol was demonstrated to enable orthodontists and surgeons to treat orthognathic patient precisely, especially during transferring the simulation into actual surgery via navigation procedures [12].

The limitations of our study are the sample size and the follow-up period. It is desirable to include sufficient samples with varied types of surgical modalities to assess the accuracy of CASNOS protocol in positioning the TMJ during orthognathic surgery. According to the study of meta-analysis by Jamilian et al., SNB showed significant increase in a 2-year follow-up while SNA and overbite increased significantly after a 2-year follow-up of the patients with skeletal class III malocclusion after bimaxillary surgery or mandibular setback. It was considered that the phenomenon was followed by residual growth of maxilla and mandible [23]. Though no obvious growth was revealed in the presurgical superimposition of the adult patient aged from 19 to 36 years (mean age 24.39 ± 4.8 years) in the present study, the long-term TMJ position brought by orthognathic surgery is to be evaluated. The other features to be investigated are the influence of different fixation methods on postsurgical position of TMJ.

## 5. Conclusions

The postsurgical position of TM joint condyles in our study presented only a mild change with the landmarks' displacement all within a range of 1.2 mm. This indicates the bimaxillary orthognathic surgery via 3D CASNOS protocol can achieve a desired and stable TMJ position in treating skeletal class III adult patients with retrognathic maxilla and prognathic mandible.

## Figures and Tables

**Table 1:** Distribution of samples by sex and age.

Sex	Amount	Mean age (years)
Male	11	24.9 ± 4.5 years (range: 20.3~33.5 years)
Female	12	4.9 ± 4.5 years (range: 20.5~33.3 years)
Total	23	24.4 ± 4.8 years (range: 20.3~33.5 years)

**Table 2:** Cephalometric measurements at 3 weeks before surgery and 2 days immediately postsurgically.

Measurement	Mean value ± SD (before surgery)	Mean value ± SD (immediate after surgery)
SNA	79.31 ± 1.52°	83.72 ± 1.28°
SNB	86.12 ± 1.50°	81.13 ± 1.32°
ANB	−6.23 ± 1.91°	2.33 ± 1.54°
GoGn-SN	31.91 ± 3.22°	34.34 ± 4.91°
Gonial angle	127.21 ± 4.15°	127.23 ± 4.23°
A-Nv	0.47 ± 1.59 mm	2.5 ± 1.2 mm
Pog-Nv	10.65 ± 3.73 mm	1.25 ± 0.58 mm
Wits	−11.81 ± 3.34 mm	1.32 ± 3.22 mm

S: sella; N: nasion; point A: subspinale; point B: supramentale; SNA: sella-nasion-point A angle; SNB: sella-nasion-point B angle; ANB: point A-nasion-point B angle; Go: gonion; Gn: gnathion; GoGn-SN: mandibular plane-SN angle; gonial angle: Ar-GoGn angle; Ar: articulare; Nv: the line goes through N and is perpendicular to the FH plane; FH plane: the plane from Po (porion, the most superior positioned point of the external auditory meatus) to Or (orbitale, the lowest point on the inferior rim of the orbit); A-Nv: the distance from point A to the Nv line; Pog-Nv: the distance from Pog to the Nv line; Wits: the distance from AO to BO (the points of contact of the perpendicular line from points A and B onto the occlusal plane are defined as AO and BO).

**Table 3:** The distance of bony movements by the surgery.

Side	Maxillary advancement (mm)	Mandibular setback (mm)
Left	3.39 ± 1.47 mm (range: 1~5 mm)	7.04 ± 2.36 mm (range: 3~13 mm)
Right	3.67 ± 1.68 mm (range: 1~6.5 mm)	5.87 ± 2.51 mm (range: 2~11 mm)

**Table 4:** The displacement of the most lateral and medial condylar points and the variation of intercondylar angles.

Parameter		
Condylar landmarks	Mean displacement (mm)	
RL-RL′	1.04 ± 0.42 mm	
LL-LL′	1.19 ± 0.41 mm	
RM-RM′	1.03 ± 0.39 mm	
LM-LM′	0.96 ± 0.39 mm	
Intercondylar angles	Mean value ± SD	*p* value
Presurgical	161.61 ± 5.08°	
Postsurgical	159.28 ± 4.92°	0.061

## Data Availability

The 3D image data used to support the findings of this study are included within the article.
